# Culinary Spices in Food and Medicine: An Overview of *Syzygium aromaticum* (L.) Merr. and L. M. Perry [Myrtaceae]

**DOI:** 10.3389/fphar.2021.793200

**Published:** 2022-01-17

**Authors:** Gloria Aderonke Otunola

**Affiliations:** Medicinal Plants and Economic Development (MPED) Research Center, Department of Botany, University of Fort Hare, Alice, South Africa

**Keywords:** culinary spices (food additives for taste), clove, pharmacological, nutritional content, functional foods and nutraceuticals

## Abstract

Spices-dried aromatic parts of plants (leaves, seeds, bark, roots, rhizomes, buds, etc) used to enhance flavour, taste and colour (sensory quality) of foods, are increasingly finding other useful roles in healthcare beyond their primary use as culinary organoleptic enhancers. Several spices are currently being investigated for their potential health benefits, because of the failing efficacy, toxicity and high cost associated with conventional drugs. One such spice: *Syzygium aromaticum* (L.) Merr. and L.M.Perry [Myrtaceae] (Clove), has a multi-dimensional role in diet, medicine, functional foods and nutraceuticals, agriculture, among other industries. Peer-reviewed articles, mostly from PubMed and Google Scholar, were consulted for the purpose of this review. The nutritional and phytochemical contents, selected biological activities as well as some functional foods and beverages of clove and their uses for human health are presented. Although these observations are largely empirical, the efficacious attributes have led to their pharmacological applications in the indigenous system of medicine all over the world and bridge between food, diet and medicine. Considering the GRAS status of clove, more studies on bioavailability, accumulation, toxicity, dosage and efficacy of clove as a spice drug or functional foods in biological systems especially in humans are required. Meanwhile, clove and its products can be used as co-adjuvants in the prevention, treatment and management of chronic diseases. Further, many applications of clove in food, health, cosmetics, pharmaceutics, nanoparticles and agricultural industries are still open for investigations.

## 1 Introduction

The interest of consumers and researchers into how food and diet prevent and treat many disease conditions and promote human health has increased in recent years. The concept of food has gone beyond the supply of basic nutrients, to optimal nutrition-the capacity of food to over and above the supply of macro- and micro-nutrients, to promote health and well-being of individuals, reduce or prevent the risk of developing diseases and sometimes reverse them (Viuda-Martos et al., 2011).

A complex relationship exists between food, health, and humans. Naturally, foods provide normal growth and development, but recent trends have demonstrated that foods can also provide health benefits that co-exist with traditional medical approaches to disease treatment when fortified with phytonutrients. Foods beyond the basic nutritional functions have potential benefits to promote health, longevity and reduce the risk of diseases. A rapidly increasing area of research is that of functional foods and nutraceuticals also referred to as foods with physiological or health benefits (Myrie and Jones, 2011). The terms fortified or functional foods are used to describe extracts and whole foods that provide protective, preventive and possibly curative effects in cancer and other chronic disease progression (Litwin et al., 2018). Nutraceuticals on the other hand refer to components/extracts of food and non-food commodities taken in the medicinal forms such as tablets, capsules, powders, liquids and potions. Functional or fortified foods resemble traditional foods in their form, but provide health benefits beyond what is rendered by their nutrient components (Shahidi, 2009).

Plant foods contain many phytomedicines-bioactive compounds known as “phytochemicals.” Some groups of phytochemicals which have, or appear to have significant health potentials are carotenoids, phenolic compounds (flavonoids, phytoestrogens, and phenolic acids), phytosterols and phytostanols, tocotrienols, organosulfur compounds, and non-digestible carbohydrates (dietary fiber and prebiotics). Bioactive plant components from medicinal plants, foods, herbs, and spices are being widely examined for their ability to provide health benefits against coronary heart disease, hypercholesterolemia, high blood pressure, diabetes, inflammation, cancers, microbial, viral, and parasitic infections and play a role in supporting treatment with modern medicine (Quilez et al., 2003; Izuegbuna et al., 2019; Ozdal et al., 2021).

Spices are aromatic or pungent vegetable substances, often dried seeds, buds, fruits, roots, or barks, primarily used for flavouring, colouring or preserving food, or to mask other flavours (Dubey, 2017). In the culinary arts, it is any dried part of a plant, other than the leaves, used for seasoning and flavouring a recipe, but not used as the main ingredient. The green leafy parts of plants used in this way are considered herbs. Every other part of the plant, including dried bark, roots, berries, seeds, twigs, or anything else that is not the green leafy part, is considered a spice. Several reports of their flavour enhancement, reduction of the need for salt, sugar and fat, preservative, digestive improvement, bioactive component and health promoting uses abound in the literature.

For ages, spices have been used as seasonings, colourants and flavourings, as well as for medicinal purposes by a huge population of the world to treat several health problems such as cancer, diabetes, obesity, hepatic, renal, and cardiovascular diseases (Baselga-Escudero et al., 2017; Otunola and Afolayan, 2018; Jiang, 2019; Batiha et al., 2020). Several spices are currently being investigated for their potential health benefits, because of the failing efficacy, toxicity and high cost associated with conventional drugs.

### 1.2 The Role of Spices in Food/Diet

The use of spices and herbs (Table 1) dates back to time immemorial and transcends early civilization. In most cuisines, spices are used as adjuncts to flavour, colour, or enhance the taste of foods. Because of their strong flavours, spices are used in small quantities and therefore do not add high amount of extra calories to the diet. However, some spices have considerably high protein, fats, carbohydrates, mineral elements, vitamins, and phytonutrients/phytochemicals contents; thus making them excellent sources of bioactive compounds which contribute to the total biological activity of the whole meal, thus providing means of managing degenerative disorders and metabolic diseases (Bhathal et al., 2020).

**TABLE 1:** Uses of spices.

Dietary	Medicinal	Cosmetic	Religious
Flavouring	Antioxidants, Anticancer	Perfumes	Anointing oils
Colouring	Anti-obesity, Antidiabetic	Deodorants	Incense
Pungency	Antihelmintic, Cardioprotective	Antiseptic soaps	Purification rites
Deodorizing/masking	Antimicrobial, Insect repellent	Hair health	Rituals
Sugar replacement	Aromatherapy		
Salt replacement			
Fat reduction			

Addition of spices to foods have resulted in improved flavour, value addition, preservative effects and longer shelf-life. For example, garlic and red chilli added to butterfat improved the flavour, red chilli, fennel or clove are used for pickles, while improved storage stability of groundnut oil was effected with red chilli and cinnamon leaves (Madsen and Bertelsen, 1995). Spices are also known to enhance digestion through the stimulation of digestive (pancreatic, terminal and small intestine) enzymes and secretion of bile, thus aiding the digestion and absorption of dietary fats (Platel and Srinivasan, 2000a; Platel and Srinivasan, 2000b; Srinivasan, 2005).

The nutritional content of spices especially with regards to macro- and micro-nutrients are important and vary from spice to spice and is dependent on several factors which include the part of the plant, harvesting technique, processing method, vegetative state, and environmental conditions, amongst others (Ereifej et al., 2015). These authors reported that a study of selected spices from Jordan revealed that dry matter of spices could range from 83.6 to 92.4%; ash 4.5–10.4%; carbohydrates 4.5–31%; protein 2.9–21.2%; fat 1.7–19.7; and fibre 25.7–59.2.

Another study reported that on dry weight basis, the crude protein of spices and herbs could range from 4.6 to 22.1%; fat (ether extract) 7.5–36.0%, total carbohydrate 34.6–71.9% and free fatty acids (as percent oleic acid) were generally low indicating good storage stability, while the flavour imparting essential oils (as percent oleoresin) were fairly high and ranged from 0.1 to 5.2% (Achinewhu et al., 1995). These data indicate that spices can contribute nutrients to the diet.

Spices are also very important in food preservation and safety. They help to eliminate the risk of food spoilage caused by lipid oxidation and spoilage microbes. Polyphenolic compounds in spices confer antioxidant properties which scavenge free radicals, chelate transition metals, quench singlet oxygen, and thus prevent oxidation in foods (Hyldgaard et al., 2012). Again, spices can prevent the growth of spoilage microorganisms (food preservation) and inhibit or regulate the growth of pathogenic organisms leading to food safety (Tajkarimi et al., 2010).

### 1.3 Medicinal Uses of Spices

Most spices along with their culinary uses, also have health-enhancing properties and have been used traditionally and culturally for the prevention, treatment and management of several diseases and ailments. The health benefits of spices are many and diverse, and range from strengthening the immune system, as nutritional supplements, control of blood sugar and cholesterol levels. In addition to these, spices have anti-inflammatory, antioxidant, anti-obesity properties and can help in preventing other diseases like mental conditions, cancer and other chronic conditions (Gottardi et al., 2016). The therapeutic effects of several spices including *Zingiber officinale* Roscoe (ginger), *Capsicum frutescens* L. (cayenne pepper), *Cinnamomum verum* J. Presl (cinnamon), *Piper nigrum* L. (black pepper), *Curcuma longa* L. (turmeric), *Trigonella foenum-graecum* L.(fenugreek), *Salvia rosmarinus* Spenn. (rosemary) and *Allium sativum* L. (garlic) against several communicable, non-communicable, and chronic diseases have been reported (Tapsell et al., 2006; Dearlove et al., 2008; Kaefer and Milner, 2008; Panickar, 2013; Opara and Chohan, 2014: Jiang, 2019; Otunola and Afolayan, 2020). The specific biological activities that support human health in spices are attributed to the presence of phytonutrients/phytochemicals or bioactive compounds which have high antioxidant effects. Several spices and herbs and their bioactive compounds (Table 2) have and are currently undergoing extensive studies to evaluate and validate their beneficial therapeutic effects (Tapsell et al., 2006; Kochhar, 2008; Islam et al., 2013; Srinivasan, 2013; Otunola and Afolayan, 2015; Baselga-Escudero et al., 2017; Jiang, 2019).

**TABLE 2:** Some spices and their major bioactive components.

Spice	Bioactive components
Rosemary	Carnosic acid, carnosol, rosemarinic acid, and rosmanol
Sage	Carnosol, carnosic acid, rosmanol, and rosmarinic acid
Oregano	Derivatives of phenolic acids, flavonoids, and tocopherols
Thyme	Carvacrol, thymol, *cymene,* caryophyllene, carvone, and borneol
Marjoram	Flavonoids
Garlic	Allicin, alliin
Ginger	Zingiberol, gingerol, zingiberene, and shogaol
Pepper	Capsaicin, vitamin C, D, and carotenoids

*Syzygium aromaticum* (L.) Merr. and L. M. Perry (clove) is as a common aromatic spice with wide global usage in cuisines and alternative medicine. In traditional and folkloric medicine, the oil is use in dentistry because of its strong analgesic and antiseptic properties (Pulikottil and Nath, 2015; Devkota and Adhikari-Devkota, 2020). Extracts and essential oil of clove has wide applications medicinally as anti-cancer, antimicrobial, anti-halitosis, anti-diabetic, anti-obesity, anti-inflammatory; antioxidant, antiviral, aphrodisiac, amongst other uses (Hussain et al., 2017; Salama et al., 2018; Kaur and Kaushal, 2019; Batiha et al., 2020; Saeed et al., 2021).

This review aimed at collating the nutritional and pharmacological importance of clove an important culinary spice as a typical example of spices (foods) at the interface of diet and medicine specifically, to show that food can be medicine. The specific objective was to collate information on the main uses and functional properties of clove, culinary and medicinal (traditional and modern) and to highlight studies reporting these functionalities. It is a summary of some of the major culinary, medicinal and functional properties of *Syzygium aromaticum* (L.) Merr. and L. M. Perry (clove) and includes a section on functional foods and nutraceuticals containing clove.

## 2 Methods

Information for this review was retrieved from repositories and search engines including PubMed, Google Scholar and Science Direct. Studies and reviews related to the culinary, nutritional and health benefits of clove, with emphasis on antioxidant, antimicrobial, anticancer, anti-diabetes, anti-inflammatory and anti-obesity potentials as well as functional foods from clove, published from 1995 to 2021, in peer-reviewed journals and reviews were retrieved. The search terms included *Syzygium aromaticum*, clove, phytochemical, culinary, pharmacological, anti-cancer, antimicrobial, anti-obesity, anti-diabetes, functional foods from clove.

### 2.1 *Syzygium aromaticum* (L.) Merr. and L. M. Perry [Myrtaceae]

Clove are the unopened flower bud of the clove tree of the family Myrtaceae. It is a species native to Indonesia and is used as a culinary spice globally (Kunnumakkara et al., 2009; Sachan et al., 2018). It is native to the Molucanna Islands (Indonesia), but now widely cultivated in many tropical regions like Zanzibar, Madagascar, Pakistan, India and Sri Lanka for commerce. As far back as 300 BC, Indonesia was the focus of Arab, Chinese, Indian and European traders for the major Indonesian spices of the time especially cloves (*Syzygium aromaticum* (L). Merr. and L. M. Perry) and nutmeg (*Myristica fragrans* Houtt.) (Sihotang et al., 2018). Historically, these spices were the cause for many exploration and expansion during the 15th century because they were highly sought after, caused great competition on finding the most efficient navigational route and conflict among those who wanted to monopolize the spice trade (https://mrlambertchemistry.wordpress.com/; Sihotang et al., 2018). A recent review (Haro-González et al., 2021) of Clove (*Syzygium aromaticum* L. Myrtaceae) essential oil revealed that the essential oil has numerous biological activities and wide application relevant to human and animal health, cosmetic, medical, flavouring, pesticide and food industries.

### 2.2 Scientific Classification

Clove belongs to the Kingdom: Plantae; Division: Magnoliophyta; Class: Magnoliopsida; Order: Myrtales; Family: Myrtaceae; Genus: Syzygium; Species: S. aromaticum (Clove); Name: Syzygium aromaticum (L.) Merr. and L. M. Perry (Bhowmik et al., 2012; Rahajaan, 2018; Saeed et al., 2021).

### 2.3 Description

Sujanapal and Kunhikannan (2017) described *Syzygium aromaticum* as a small evergreen tree, 6 m tall, conical crown, leaves 7–12 × 3–5 cm, elliptic or oblanceolate, base attenuate or cuneate, apex acuminate, margin entire, glabrous, coriaceous, punctate beneath; lateral nerves many, parallel, obscure, with intramarginal nerve; petiole 10–20 mm long, slender, glabrous. The flowers are pinkish-white, fragrant, in 4 cm long cymes; calyx 1.5 × 6 mm, tubular, verrucose with four hook-like involute ascending segments; petals up to 10 × 5 mm, elliptic, calyptrate; stamens many, inflexed in the bud; ovary inferior. Berry globoid or ellipsoid, dark purple with persistent calyx ring on the top. The flower buds are of commercial value as spice.

### 2.4 Nutritional Content and Culinary/Dietary Uses of Clove

Clove belongs to the generally regarded as safe (GRAS) foods list. It is used to flavour foods and beverages especially rice, masala and soups. Nutritionally, 100 g of clove contain 66 g carbohydrates, 6 g protein, 13 g fat and has 274 calories, as well as rich in vitamins A and B6; iron, calcium, magnesium and phosphorus (https://www.wildturmeric.net/clove-health-benefits-medicinal-uses/). Clove spice is pungent and astringent, enhance circulation, digestion and metabolism as well as alleviate stomach disorders (Dey and Mukherjee, 2021). Nutritionally, 100 g of ground clove is reported to contain Water 5.40–6.86 g; Food energy 323 (Kcal); Protein 5.98 g; Fat 20.06 g; Carbohydrate 61.22 g; Ash 5.88 g; Ca 0.646 g; P 105 mg; Na 243 mg; K 1102 mg; Fe 8.68 mg; Thiamin 0.115 mg; Riboflavin 0.267 mg; Niacin 1.458 mg; Ascorbic acid 80.81 mg; Vitamin A 53 RE (Tainter and Grenis, 1993; Dey and Mukherjee, 2021). Clove has been used in some food formulations to enhance the nutritional, preservative and biological activities of such products. Some of these are highlighted.

A study on the sensory, antioxidant, and maillard reaction profiles of rye-buckwheat cakes enhanced with selected spices, which included clove revealed that the phenolic content, antioxidant capacity, browning properties, and overall acceptability of cakes enriched with clove, allspice, and spice were elevated and best (Przygodzka et al., 2015). According to Schlieck et al. (2021), addition of a blend of essential oils of clove, rosemary and *Origanum vulgare* L. (oregano) and vitamin E to replace conventional chemical antioxidants in dog feed improved the food quality and health of beagles considerably. Another study used and compared clove, ginger and *Cymbopogon citratus* (DC.) Stapf (lemon grass) as flavourants in cakes. The outcome revealed that clove spiced cake had a very nice texture, taste and nutrients and was best in terms of acceptability by consumers (Forlemu and Amadou, 2021). Other culinary uses of clove are as studding for *Allium cepa* L. (onions), *Solanum lycopersicum* L. (tomatoes), salads, herbal teas, and soups, to flavour meat products, cookies, pastries, sandwiches, pickles, puddings, chewing gums, spiced fruits, chocolates, soft drinks and candies (Hussain et al., 2017).

### 2.5 Metabolites of Clove

Several studies have investigated and reported on the metabolites present in the essential oil and various extracts of clove.

Alma et al. (2007) reported the presence of 18 components in clove bud essential oil, and the most abundant was eugenol (87%), chavibetal (19.7%), β-caryophyllene (13%). A GC-MS analysis of the essential oil of clove by El-Ghallab et al. (2020) revealed a total of 13 bioactive compounds of which Eugenol, β-Caryophyllene, eugenyl acetate and α-Humulene were the most abundant. Several other authors have reported varying abundance of phytochemicals for clove essential oil, but all pointed to the fact that eugenol was the major bioactive component (Viuda-Martos et al., 2007; Hussain et al., 2017; Shahidi and Hossain, 2018; Sgorbini et al., 2015). Other bioactives present in clove are kaempferol, quercetin and its derivates, caffeic, ferulic, elagic, and salicylic acids, as well as other minor constituents like α-humulene, β-humulene, methyl salicylate, crategolic acid, and benzaldehyde, that account for the characteristic pleasant fragrance of clove (Hussain et al., 2017). Six sesquiterpenes, α-cubebene (1.3%), α-copaene (0.4%), β-humulene (9.1%), β-caryophyllene (64.5%), γ-cadinene (2.6%) and δ-cadinene (2.6%) have been characterized from the hydrocarbon fraction of the freshly distilled Indian clove bud oil (Gopalakrishnan et al., 1984).

Nassar et al. (2007) evaluated the chemical constituents of clove (*Syzygium aromaticum*, Fam. Myrtaceae) using GC-MS, chemical and spectroscopic methods including 1D and 2D NMR for identifications of the compounds. They reported sixteen volatile compounds in the n-hexane extract with eugenol (71.56%) and eugenol acetate (8.99%) as the major components; limonin, ferulic aldehyde, and eugenol in the dichloromethane extract; tamarixetin 3-O-b-D-glucopyranoside, ombuin 3-O-b-D-glucopyranoside and quercetin from the ethanol extract. They also reported the hepatoprotective activity of the ethanol extract against paracetamol-induced liver injury in female rats.

Hemalatha et al. (2016) subjected the methanol, acetone and chloroform extracts of clove to phytochemical analysis and confirmed the presence of phenols, flavonoids, alkaloids, tannins, and saponins. The dichloromethane extract of clove bud oil showed the presence of carbohydrates, terpenoids, glycosides, steroids, sterols, tannins, and phenolic compounds (Kumar et al., 2010); some other studies of methanol extract reported saponins, alkaloids, flavanoids, cardiac glycosides, tannins, and steroids and forty-six phenolic compounds in methanol extract of clove (Jimoh et al., 2017; Kaur and Kaushal, 2019). Clove ethanolic extract contained fixed oil, tannins, phenolic compounds, terpenoids, cardiac glycosides but no saponin (Rosarior et al., 2021).

### 2.6 Medicinal and Pharmacological Importance of Clove

Historically, clove have been used in Ayurveda, Chinese and Western traditional medicine as a painkiller for dental emergencies (Daniel et al., 2009; Sachan et al., 2018). The antimicrobial, antioxidant, antiviral, anaesthetic, antiparasitic, antioxidant action, antiperspirant action, antiseptic property, carminative action, deodorant, digestive disorders, rubefacient, immune-boosting, stomachic actions Alzheimer’s disease, among many others have been reported (Sujanapal and Kunhikannan, 2017; Sachan et al., 2018). Most of the medicinal and pharmacological properties attributed to clove are premised on its various phytochemical and bioactive constituents especially eugenol. The medicinal and pharmacological importance of clove essential oil and extracts are numerous and while transcending its dietary role, acts/points to the important link between food and medicine.

### 2.7 Antioxidant Activity

Clove oil has been accredited with high antioxidant activity, perhaps one of the best known oil for food or supplement, which is attributed to the phenolic compounds especially eugenol, thymol and eugenol acetate (Yadav and Bhatnagar, 2007; Dai et al., 2013; Nam and Kim, 2013; Mittal et al., 2014; Uddin et al., 2017; Kaur and Kaushal, 2019). The high antioxidant capacity of eugenol has been compared to that of pyrogallol and BHT. According to Fankem et al. (2017) clove essential oil exhibited ten times higher antiradical activity than BHT; and seven times greater than cocoa butter and clove essential oil mixture. When a linoleic acid emulsion was treated with 15 μg/ml of clove oil, 97.3% inhibition of lipid peroxidation was observed compared to inhibition by standard antioxidants like trollox, BHA, α-tocopherol and BHT which showed 95.6, 95.4, 84.6, and 99.7% inhibition respectively (Gulcin et al., 2012). Clove ethanolic extract exhibited dose-responsive antioxidant effect and better reducing potential, against DPPH, ABTS, and Ferric chloride respectively (Rosarior et al., 2021). Clove and lavender (*Lavandula angustifolia* Mill.) ethanol and aqueous extracts at 20, 40, and 60 μg/ml showed inhibitions up to 95% when tested as superoxide radical capture, scavenging of DPPH radical and as metal quelants (Cortés-Rojas et al., 2014). The powerful antioxidant activity of both extracts were attributed to strong hydrogen donating, metal chelating and free radical scavenging capacity.

Clove extract was also reported to exhibit antioxidant properties when tested using ferric reducing antioxidant power, oxygen radical absorbance capacity, DPPH, hydroxyl and superoxide radicals scavenging activities (Suantawee et al., 2015). According to Chatterjee and Bhattacharjee (2015) Mayonnaise formulated with eugenol-lean clove extract showed distinctly higher antioxidant activity and reducing power than mustard-formulated mayonnaise and the market sample.

### 2.8 Assessment

Although clove extracts and oil have been credited with very high antioxidant capacity, the antioxidant activities reported here are mostly “*in vitro*”, which according to Gafner (2018) is present in all plants and as such not useful without additional data. Moreover, the tests (ABTS, ORAC, DPPH, TEAC, NO, and FRAP^†^ assays) used for measuring total antioxidant activity, are non-specific, prone to interferences and the results are often unreliable; also the assurance that *in vitro* antioxidant activities will be observed in animal or human studies is uncertain.

### 2.9 Antimicrobial Activity

#### 2.9.1 Antibacterial

Yassin et al. (2020a) evaluated the antibacterial efficiency of acetone, dichloromethane, ethanol and petroleum ether extracts of clove against *Escherichia coli, Salmonella typhi, Staphylococcus aureus* and Methicillin-resistant *Staphylococcus aureus* (MRSA). The researchers reported that the dichloromethane extract exhibited the highest antibacterial potency against the pathogenic isolates.

A study by Radünz et al. (2019) showed that unencapsulated and encapsulated clove essential oil at 0.304 mg/ml inhibited *S. aureus*, *E. coli*, *L. monocytogenes*, and *S. Typhimurium* bacteria, independent of the microorganism membranes and *in situ* inhibition against *S. aureus* in meat products similar to burgers. The bactericidal action of clove essential oil is attributed to eugenol, which ruptures the bacterial cytoplasmic membrane, allowing leakage of ions and intracellular proteins, leading to cell death.

The high antibacterial effect of ethanol and methanol extracts of clove against *Escherichia coli*, *Pseudomonas aeruginosa* and *Klebsiella pneumoniae* clinical isolates have been reported (Garba et al., 2019). Ethanolic extract of clove was shown to have broad spectrum (Gram-positive and Gram-negative) inhibition on five pathogens causing urinary tract infections, this inhibition was stronger than that shown by pure eugenol at the same concentration (Rosarior et al., 2021). Antibacterial activity of methanol extract was reported for *Klebsiella pneumonia* (Hemalatha et al., 2015).

Eugenol, the major component of clove oil was shown to have bactericidal action against *Proteus mirabilis* at the minimum inhibitory concentration of 0.125% (v/v) and at 0.25% (v/v) it reduced viability and completely inhibited *P. mirabilis* population within 30 min of exposure (Devi et al., 2013).

Another study showed that hexane extract of clove (*Eugenia caryophylata*), at 0.2% exhibits the highest inhibition against the pathogenic potato black stem and soft rot bacteria, *Erwinia carotovora subsp. atroseptica* when compared to ethanol extracts of cinnamon (*Cinnamomum zelanicum*) and datura (*Datura metel*) at the same concentration (Al-Jeboory et al., 2012).

#### 2.9.2 Antifungal

Yassin et al. (2020b) showed that *Syzygium aromaticum* (clove) ethyl acetate extract exhibited the highest antifungal activity against *C. tropicalis*, *C. albicans, C. glabrata,* and with minimum inhibitory concentration of 250 μg/disc and 500 μg/disc respectively, while the minimum fungicidal concentration was 0.5 mg/disc against *C. tropicalis* and 1 mg/disc against the *C. albicans* and *C. glabrata.* The main bioactive compounds in the extract were eugenol (58.88%), eugenyl acetate (23.86%), *trans*-caryophyllene (14.44%) and α-humulene (1.88%).

Clove oil was reported to show maximum antifungal activity against *C. tropicalis, C. albicans,* and *C. guilliermondii* (Kumar et al., 2012). Encapsulated clove oil also exhibited strong antifungal action against *Fusarium oxysporum* (Estrada-Cano et al., 2017) and antiseptic effects on meat products at a concentration above 0.070% (Wang et al., 2018; Kaur and Kaushal, 2019).

#### 2.9.3 Antiviral Activity

Aqueous extract of clove was reported to exhibit a dose-dependent antiviral effect against Feline Calicivirus, used as a surrogate for Human Norovirus, a food-borne virus. The effect was attributed to the main bioactive component-eugenol which showed similar antiviral activity to clove extract, although at lower level, an indication that other components in clove worked synergistically with eugenol to inactivate Feline Calcivirus (Aboubakr et al., 2016).

Eugenol and Eugenin isolated from clove bud essential oil was also shown to have potent inhibitory effect against herpes simplex virus at a dose of 10 μg/ml (Milind and Deepa, 2011). The antiviral efficacy of aqueous extracts of clove against herpes simplex virus type 1 (HSV-1) and influenza A virus when combined with acyclovir has also been reported (Batiha et al., 2020).

#### 2.9.4 Assessment

According to the above mentioned studies, both the extracts and essential oil of clove possess antimicrobial activity. Most of these activities were dose dependent, broad spectrum and based on disruption of the microbial cytoplasmic membrane resulting in leakage of cytoplasmic content and subsequent cell death. However, most of the studies did not give the mechanism or type of inhibition, but all reported that clove can be used as a natural antibacterial, antifungal and antiviral agent in human/animal health, as well as the inhibition of pathogenic and spoilage microorganisms in foods and plants.

### 2.10 Anti-Inflammatory Properties of Clove

#### 2.10.1 *In vitro*

Anti-inflammatory activity of clove (*Eugenia caryophyllata*) essential oil was evaluated by simulating inflamed human skin cells in a dermal fibroblast system (HDF3CGF). Clove essential oil at a concentration of 0.001% showed strong anti-inflammatory action, demonstrated through resistance to the production of pro-inflammatory biomarkers like interferon-inducible T-cell α-chemoattractant (I-TAC), vascular cell adhesion molecule-1 (VCAM-1) and collagen III expression at gene and protein levels (Han and Parker, 2017).

Bachiega et al. (2012) reported that aqueous extract of clove and eugenol in non-cytotoxic concentrations exerted immunomodulatory or anti-inflammatory action on cytokine production by murine macrophages incubated with clove or eugenol (5, 10, 25, 50 or 100 µg/well) for 24 h. They showed that 100 µg/well of clove inhibited the production of IL-1*β*, IL-6, and IL-10, eugenol did not affect IL-1*β* production but inhibited IL-6 and IL-10. They concluded that the immunomodulatory/anti-inflammatory effects of clove was by inhibiting LPS action and the possible mechanism of action may be through suppression of nuclear factor-*κ*B pathway by eugenol, because it was the major bioactive of clove extract.

#### 2.10.2 *In vivo*

Nikoui et al. (2017) investigated the anti-inflammatory and antipyretic effects of clove oil in healthy dogs after surgery. Animals surgically operated in the abdomen were grouped into four and administered 25 mg/kg of clove oil, 20 mg/kg betamethasone (anti-inflammatory), 15 mg/kg phenylbutazone (anti-pyretic), or nothing as control for 5 days consecutively. The researchers reported that clove oil-treated animals had significantly reduced edema, white blood cells, neutrophils, band neutrophils and rectal temperature compared to control. Also, histopathology assay showed that clove oil reduced inflammation significantly in dogs.

Also, the use of pure clove oil in the aromatherapy treatment of arthritis and rheumatism, in combination with honey for dermatitis and the paste (water and clove powder) for bites and cuts have been reported (Hussain et al., 2017).

Further, Naji et al. (2017) evaluated the anti-inflammatory capacity of *Syzygium aromaticum* extract (SAE) using formalin test and showed that mice fed with different doses of SAE exhibited visible increase in analgesia time and decrease in licking number (*p* < 0.05) compared to untreated animals.

Bachiega et al. (2012) showed that clove and eugenol exhibited immunomodulatory and anti-inflammatory action on cytokine production by murine macrophages when administered in non-toxic doses. In addition, clove oil at 0.05 and 0.20 ml/kg showed anti-inflammatory effect equal to that of anti-inflammatory drugs like etodolac and indomethacin at 0.025 and 0.1, and 0.05 and 0.2 ml/kg doses, respectively (Vicidomini et al., 2021).

#### 2.10.3 Assessment

The anti-inflammatory actions of clove extracts and essential oils though documented, were mostly performed using *in vitro* and *in vivo* assays using cell lines and animals respectively. No clinical study involving human subjects were reported. Many studies evaluated the anti-inflammatory potentials using eugenol the major bioactive component of clove. The studies report the potential of clove extracts and oil and provide support for its use in folk medicine. However, well planned studies across all levels (*in vitro*, *in vivo* and clinical/human) including dosages are urgently needed to validate the anti-inflammatory use of clove.

### 2.11 Anti-Cancer Activity

#### 2.11.1 *In vitro* Studies

##### Toxicity

Cytotoxicity of acetone, dichloromethane, ethanol and petroleum ether extracts of clove against human colon cancer (HCT) cell lines using MTT assay were investigated (Yassin et al., 2020a). The test revealed that ethanol extract of clove gave maximum cytotoxic potency against HCT cell line, dichloromethane extract was least effective, whereas the petroleum ether and acetone clove extracts showed only moderate cytotoxicity against HCT cells.

Another study by Kello et al. (2020) revealed that treatment of MCF-7 cells with ethanol extract of clove (CBE) induced intrinsic caspase-dependent cell death associated with increased oxidative stress mediated by oxygen and nitrogen radicals, release of mitochondrial pro-apoptotic factors, signalling of oxidative stress-mediated DNA damage with modulation of cell antioxidant SOD (superoxide dismutase) system, and modulation activity of the Akt, p38 MAPK, JNK, and Erk 1/2 pathways.

Nirmala et al. (2019) evaluated the anticancer (cytotoxicity) potential of a clove bud essential oil-based nanoscale emulsion system (CB-4) against thyroid cancer cell line (HTh-7) using MTT, colony formation and Annexin V-FITC assays. The authors reported that 0.7 μL/ml of CB-4 was cytotoxic against thyroid cancer cell lines (HTh-7) but not to non-cancerous cells (Hek-293); also significant reduction was observed in the number of cancer cell colonies when treated with CB-4 for 7–10 days, while Annexin V-FITC confirmed that the clove bud oil-based emulsion has antiproliferative action on growth of HTh-7 cell line, an indication that apoptosis may be responsible for cell death though necrosis was also observed when stained with propidium iodide.

Khan et al. (2018) tested fluorescent magnetic submicronic polymer nanoparticles (FMSP-nanoparticles) alone (1.25, 12.5, 50, 75, and 100 *μ*g/ml) and combined with crude clove extracts (50 *μ*g/ml) on human breast cancer cells (MCF-7) for 24 and 48 h, using Trypan Blue, 4′,6-diamidino-2-phenylindole (DAPI) and 3-(4,5-dimethylthiazol-2-yl)-2,5-diphenyltetrazolium bromide (MTT) assays. They showed that when treated with FMSP-nanoparticles alone, cancer cell viability was decreased to 55.40%, but decreased drastically to 8.50% when treated with combination of FMSP-nanoparticles and crude clove extracts.

Another study reported that clove essential oil at concentration of 0.011% was potent against proliferation of human dermal fibroblasts by significantly inhibiting the production of pro-inflammatory biomarkers (vascular cell adhesion molecule-1 (VCAM-1), interferon γ-induced protein 10 (IP-10), interferon-inducible T-cell α chemoattractant (I-TAC), and monokine induced by γ interferon (MIG) and tissue remodelling protein molecules, namely, collagen-I, collagen-III, macrophage colony-stimulating factor (M-CSF), and tissue inhibitor of metalloproteinase 2 (TIMP-2); as well as modulation of gene expression and altering signalling pathways critical for inflammation, tissue remodelling and cancer signalling processes (Han and Parker, 2017).

The *in vitro* antitumor effects of clove ethyl acetate extract and the bioactive component (oleanoic acid) responsible for the activity was investigated (Liu et al., 2014). The ethyl acetate extract and oleanoic acid were reported to be cytotoxic, inhibit tumor growth, promote G_0_/G_1_ cell cycle arrest and apoptosis in several human cancer cell lines in a dose-dependent manner, selectively increased protein expression of p21^WAF1/Cip1^ and γ-H2AX and downregulated expression of cell cycle-regulated proteins.

Anti-proliferative activity of aqueous, ethanol and oil extracts of clove against HeLa (cervical cancer), MCF-7 (ER + ve) and MDA-MB-231 (ER—ve) breast cancer, DU-145 prostate cancer, TE-13 esophageal cancer cell lines and normal human peripheral blood lymphocytes was investigated. The results showed that clove oil at 300 μl/ml caused maximum (80%) and apoptotic cell death in TE-13 cells within 24 h, but minimal cell death in DU-145 cells with no significant cytotoxicity in human PBMC’s at the same dose (Dwivedi et al., 2011). β-caryophyllene had no cytotoxic effect, but clove oil and eugenol were cytotoxic. They proposed the possibility that natural compounds found in clove could be used for the development of new treatment for esophageal cancer.

#### 2.11.2 *In silico* Studies

Azadi et al. (2020) using *in silico* mathematical modelling and 1H Nuclear Magnetic Resonance spectroscopy on clove oil treated Raji cells, used the Metaboanalyst software to predict that 50% inhibitory concentration of clove oil was 50 μg/ml and 74 genes with differentiating metabolites consisting of amino acids, cholesterol and fucose. They also predicted that clove oil mechanism of anti-cancer action was against novel enzymes, like 24-dehydrocholesterol reductase and 7-dehydrocholesterol reductase in cholesterol biosynthesis, dehydrofolate reductase in one carbon metabolism and serine palmitoyl-transferase long chain in sphingolipid biosynthesis.

#### 2.11.2 *In vivo* Studies

Kubatka et al. (2017) showed that dietary administration (0.1 and 1%) of clove was effective against induced mammary carcinoma in rat model through significant decrease in tumor frequency but not latency. The antitumor effect was mediated through caspase three activation, reduction of protein (Bcl-2, Ki67, vascular endothelial growth factors A, MDA, CD24, CD44) expressions and increased methylation of RASSF1A promoter, as well as expressions of ALDH1A1, H4K16ac, and H4K20me3. Colon cancer was induced in mice by injecting them with naked HT-29 tumor cells, the mice were then treated with ethyl acetate extract of *S. aromaticum* once daily for 5 days. The authors reported that tumor growth was significantly reduced in the extract-treated group (Kubatka et al., 2017).

*In vivo*, the antitumor activity of ethyl acetate extract and oleanoic acid from clove were investigated using the HT-29 tumor xenograft model (Liu et al., 2014). The extract showed higher activity than oleanoic acid (one of its bioactive components) and 5-fluorouracil (a chemotherapeutic agent) at suppressing the growth of colon tumor xenografts. Most of the changes reported were at the mRNA level, suggesting transcriptional regulation by ethyl acetate extract. The authors concluded that clove extract and oleanoic acid, could be novel therapeutic agents for the treatment of colorectal cancer.

#### 2.11.3 Assessment

From the studies reviewed, clove extracts, essential oils and bioactive components exerted anti-cancer properties *in vitro* through cytotoxicity, apoptosis, chemopreventive, tumor formation, antiproliferative, modulation of gene expression and cancer signalling processes actions. *In silico* evaluation have also been used to predict anti-cancer potential of clove. *In vivo*, clove extracts inhibited tumor growth through caspase three activation, gene and protein expressions. Unfortunately, as with anti-inflammatory action of clove, no human studies have been documented so far. Thus it is pertinent that future studies be initiated to investigate the anticancer effect of clove in humans including the dosage. Presently though, since clove is a GRAS food, it can be used as an adjuvant in cancer treatment.

### 2.12 Anti-Diabetic Activities

The anti-diabetic effects of clove essential oil and extracts are well documented. It is assumed that clove extract mimics the action of insulin on gene expression of glucose-6-phosphatase and phosphoenolpyruvate carboxykinase; and that expression of several genes are regulated by clove and insulin in the same pattern (Milind and Deepa, 2011).

### 2.12.1 *In vitro*

Adefegha and Oboh (2012) reported that polyphenol-rich extracts from *Syzygium aromaticum* (L.) Merr. and Perry (Clove) buds inhibited the activity of carbohydrate hydrolyzing enzymes linked to type 2 diabetes and Fe2+-induced lipid peroxidation in rat pancreas in a dose-dependent manner *in vitro*. The authors reported that alpha-glucosidase inhibition was significantly higher than that of alpha-amylase and could be the mechanism by which clove elicits exerts its ameliorative effect on type 2 diabetes.

### 2.12.2 *In vivo*

Dietary intake of clove by streptozotocin-induced diabetic male Sprague-Dawley rats led to reduced tissue and cardiac muscle damage, reduced blood sugar and lipid peroxidation, decrease in hyperglycaemia-induced oxidative tissue damage and cataract formation in the eye lens (Lakshmi and Manasa, 2021).

Hydroalcoholic extracts of clove reportedly improved serum biomarkers, antioxidant status, and histopathological changes in kidneys of streptozotocin-induced diabetic rats by ameliorating glycaemic control, lipid profile and preventing kidney damage (Abtahi-Eivari et al., 2021).

Sammy et al. (2020) reported that when streptozotocin induced diabetic rats were administered aqueous extract of clove at 250 mg/kg body weight with metformin, glucose, ALT, AST, and other biomarkers were significantly downregulated compared to the untreated or no clove streptozotocin treated diabetic rats. Also, Abdulrazak et al. (2018) showed that high fat diet-induced type 2 diabetic rabbits treated with 12.5% dietary clove and 12.5% fermented ginger respectively for 6 weeks exhibited significant (*p* < 0.05) decrease in blood glucose levels and that the clove supplement effectively sustained anti-hyperglycemic activity, lower effect on leptin levels, with significant increase in insulin levels compared to diabetic control group.

According to Abd El-Rahman (2015) dietary inclusion of *Syzygium aromaticum* at 3, 5, 7 and10% for 4 weeks in the diet of alloxan-induced diabetic rats, caused significant decrease in serum glucose, total cholesterol, LDL-C, VLDL-C, triglyceride levels, AST, ALT, and ALP, as well as liver function and lipid profile.

Adefegha et al. (2014) reported that induced type 2 diabetes rat model fed on diets containing clove bud powder at 20–40 g kg−1 exhibited reduced blood glucose level compared to control diabetic rats without clove bud powder supplementation; in addition, reduced α-glucosidase and liver enzymes activities along with elevated levels of antioxidant status were observed.

Aqueous extracts of cinnamon and clove at 10 μg/ml showed very strong and potent inhibition of protein glycation in zebra fish (Jin and Cho, 2011).

#### 2.12.3 Clinical

Bi et al. (2017) reported a study in which 36 people with T2D were given capsules containing 0, 1, 2, or 3 g of cloves per day for 30 days and a 10-days washout period. Results from the study showed that although no significance differences in responses to the dose was observed, diabetic patients who took clove supplement exhibited decrease in serum glucose, total cholesterol, triglycerides and LDL, compared to diabetic patients who did not.

### 2.13 Anti-Obesity Activities

#### 2.13.1 *In vitro*

An alcoholic extract of clove (containing majorly-eugenol (42.27%), acetyl eugenol (29.12%), caryophyllene (15.40%), and humulene (3.22%) was reported to inhibit S-phase DNA replication of HepG2 cells and adipocyte differentiation of OP9 cells (Ding et al., 2017). *In vitro* treatment using clove ethanol extract on 3T3-L1 cells showed that the extract efficiently inhibited the conversion of cells into adipocytes in a dose-dependent manner (Jung et al., 2012).

#### 2.13.2 *In vivo*

Ding et al. (2017) reported that an alcohol extract of clove [containing majorly-eugenol (42.27%), acetyl eugenol (29.12%), caryophyllene (15.40%), and humulene (3.22%)] prevents obesity in a mouse model by acting as a natural fatty acid inhibitor. The extract also reduced the development of high fat diet-induced obesity, body and abdominal adipose tissue weight, regulate total triglyceride, low-density lipoprotein cholesterol and lower lipid accumulation in the liver and epididymal adipose tissues.

In another study, Jin and Cho, (2011) showed that an aqueous extract of cinnamon and clove at 10 μg/ml administered to hyper-cholesterolaemic zebrafish exerted a potent dose-dependent cholesteryl ester transfer protein (CETP) inhibitory and hypolipidaemic activities; and that clove extract-fed group had the smallest increase in body weight and height and the strongest antioxidant activity after a 5-weeks high cholesterol diet.

Another study reported that clove ethanol extract reduced diet-induced obesity in mice fed high fat diet supplemented with 0.5% (w/w) for 9 weeks through down-regulation of adipogenic and lipogenic gene expression (Jung et al., 2012). Another study reported that simultaneous administration of clove and curcumin (*Curcuma longa* L.) extract on high fat diet-induced mice for 5 weeks led to reduced food intake, weight gain, adipose tissue, liver weight, and regulation of lipid profiles (Pérez Gutiérrez and Arrioja, 2021).

#### 2.13.2 Assessment

Regarding antidiabetic and anti-obesity potential of clove, most of the studies were on extracts and essential oils of clove and were mostly *in vivo* using mice or rat models and *in vitro* using cell lines and enzyme based assays. The activities were marked with reduction in fasting blood glucose, protein glycation, increased insulin production, improving glucose tolerance, and glucose and lipid metabolism-related enzyme activities and conversion of cells into adipocytes among other physiological actions. Only one clinical study in humans was reported for antidiabetic property of clove. These studies however, are not detailed enough, neither are they supported with human studies considering the GRAS status of clove.

Therefore, future studies should focus on the effect of clove on humans through randomized clinical trials, targeting specific biomarkers to confirm and validate the folkloric uses of clove as therapy for diabetes and obesity.

#### 2.13.3 Functional foods and Nutraceuticals prepared with clove.

Paschoalinotto et al. (2021) showed that a tisane (T5) composed of lemon thyme, tutsan, cloves and cinnamon, exhibited health-promoting effects (lipid peroxidation inhibition, anti-inflammatory, and anti-diabetic activities) and potential for application in the food and nutraceutical industries.

In another study, Günes-Bayir et al. (2020) investigated the effect of adding clove (*Syzygium aromaticum*) in three different concentrations (0.1, 0.3, and 1%) and propolis (0.03%) to probiotic yoghurt as follows: 1) yoghurt without propolis or clove (control group); 2) yoghurt with propolis (0.03%); 3) yoghurt with propolis (0.03%) and clove (0.1%); 4) yoghurt with propolis (0.03%) and clove (0.3%); 5) yoghurt with propolis (0.03%) and clove (1.0%) respectively on the microbiological, chemical and sensory properties of probiotic yoghurts. They reported that whereas propolis exerted antibacterial effect on bacteria colonies except *Streptococcus thermophiles*, clove reduced the suppressive effect of propolis on bacteria colonies by supporting the number of *B. animalis subsp. lactis* growth, improving the microbial qualities and sensory acceptability of the probiotic yoghurts positively.

Nirmala et al. (2019) prepared nanoscale-based emulsions from essential oil of clove buds (*Syzygium aromaticum*) and varying concentrations of Tween 20 and Tween 80 surfactants. Cytotoxicity of the stable oil-based emulsion was evaluated using MTT colony formation, and Annexin V-FITC assays against thyroid cancer cell line (HTh-7). The authors reported that the product has potential as alternative cancer candidate drug because it showed apoptotic and reductive effect on cancer cell proliferation.

Cinnamon (*Cinnamomum zeylanicum*), fenugreek (*Trigonella foenum-graecum*), shallot (*Allium hirtifolium Boiss*) and clove (*Syzygium aromaticum*) were evaluated for their antidiabetic efficacy in rats, singly and as a polyherbal formulation (Tetraherbs) in streptozotocin (STZ)-induced diabetic rats with fasting blood sugar above 350 mg/dl. The rats were treated orally with 75 mg/kg dose ethanolic extracts of each specie separately, or in equal combination formulation (Tetraherbs) at 100–300 mg/kg once daily for 28 days. Tetraherbs gave a higher blood glucose lowering activity and pancreatic β cell regeneration than for each plant (Kiani et al., 2018).

Another study by Ramos et al. (2017) evaluated an herbal extract containing green mate (*Ilex paraguariensis*), clove (*Syzygium aromaticum*), and lemongrass (*Cymbopogon citratus*) optimized in fermented milk with or without sweet potato pulp. Addition of a lyophilized extract (1 g 100 g−1) of 87.5% clove and 12.5% green mate increased the antioxidant and total phenolic content, though fermented milk with added sweet potato pulp had the best sensory acceptance. Twenty-one-day storage revealed slight decrease in total phenolic acid and total reducing capacity. The authors proposed the addition and use of this extract for the development of new dairy foods with potential functional properties.

The safety and anti-ulcerogenic activity of a Clovinol, a novel polyphenol-rich extract of clove buds (*Syzygium aromaticum* L) was reported by Issac et al. (2015). Clovinol was derived as a water soluble free flowing powder from clove buds, without the characteristic pungency and aroma. The extract was characterized by electrospray ionization-time of flight mass spectrometry (ESI-TOF-MS) and evaluated for antioxidant, anti-inflammatory and anti-ulcerogenic activities in mice and rat models. The product exhibited significant antioxidant and anti-inflammatory effects measured by cellular antioxidant levels and inhibition of carrageenan-induced paw swelling in mice; while its anti-ulcerogenic activity was confirmed by greater than 97% inhibition of ethanol-induced stomach ulcers in Wistar rats administered at 100 mg per kg b w. orally and up regulation of *in vivo* antioxidants. Lipid peroxidation, oxidative stress and gastrointestinal health of ulcer induced rats were also improved by Clovinol, which was shown to be safe at 5 and 2.5 g per kg body weight for acute and 28 days of repeated dose toxicity studies respectively.

Babajide et al. (2013) blended equal portions of cucumber (50%) and pineapple (50%) juices with clove and ginger powder at 0.25% (CPCLG1), 0.5 (CPCLG2), 0.75% (CPCLG3), and 1% (CPCLG4) (w/v) respectively to develop a new fruit drink with health benefits. They reported the presence of alkaloids, flavonoids, saponins, steroids, tannins, terpenoids and phlobatannins. Various Quantities of isoquinoline, octanoic acid, metroprolol, fumaric acid, benzoquinone, and betaxolol among others.

Balaji et al. (2012) investigated the antidiabetic potentials of a novel polyherbal preparation containing *Asparagus racemosus, Emblica officinalis, Salacia oblonga, Syzygium aromaticum,* and *Tinospora cordifolia* in equal ratios. The authors showed that the product reverted the activities of glycolytic and gluconeogenic enzymes in diabetic rats and that lipid profile, antioxidant status and glycogen content were improved, with decrease in lipid peroxidation.

#### 2.13.4 Assessment

The studies reported here are some examples of the use of clove in preparation of functional foods and beverages, tailored towards the management of some chronic diseases. Some of these show that the therapeutic property of clove can withstand food processing/thermal changes and are carried through to the final products. However, more studies are needed to determine appropriate vehicles, doses, shelf-life and toxicity/adverse consequences in human trials to validate these therapeutic claims. These functional foods however, can be used co-adjuvantly in the prevention, treatments and management of chronic diseases.

## 3 Conclusion, Limitations, and Future Research Needs

The traditional, historical and cultural use of spices in diet and medicine cannot be overemphasized, because along with their culinary uses, most spices also have health-enhancing properties as demonstrated by clove (*Syzygium aromaticum* (L.) Merr. and L. M. Perry [Myrtaceae]).

Traditionally and pharmacologically, the medicinal uses of clove spice are diverse and seemingly unending. This review collated available information on nutritional and phytochemical constituents, forms (powders, extracts, infusions, and combination with other spices, etc.), concentrations/dosages, selected biological/health activities, as well as functional foods derived from clove spice were considered.

Some of these include use of clove oil for toothache, dental caries and pyorrhea; headache, sore throat, respiratory disorders (coughs, colds, bronchitis, asthma, sinusitis, and tuberculosis) and digestive system ailments. In traditional medicines of Australia and Asian countries, clove is used for ear ache, anti-inflammatory, analgesic, antipyretic, antifungal, antibacterial and peptic ulcer treatments. Other uses include as an anaesthetic, hepatoprotective, anthelmintic, memory recall, anti-stress, analgesic, anticonvulsant, antimycotic, insecticidal, antimutagenic, and antiulcerogenic amongst other uses (Milind and Deepa, 2011; Agrawal et al., 2018; El-Ghallab et al., 2020; Yadav et al., 2020).

This overview has revealed documented scientific evidence that clove spice possesses antioxidant, anti-microbial, anti-viral, anti-inflammatory, anti-cancer, anti-diabetic and anti-obesity functions amongst other reported properties. However, this could be further buttressed through clinical studies.

Thus, the medicinal and pharmacological importance of spices are an excellent validation of the common statement “Let food be thy medicine and medicine thy food; ’’ and the link between food and medicine.

The review was limited by the fact that so many of the studies reported were *in vitro* with very few clinical studies. Most of the studies also did not report the percentage of effectiveness of the extract or oil and the mechanism of action.

Future research needs and focus should be on well-planned and executed studies on the medicinal properties of clove using different models including *in vitro, in vivo, ex vivo,* and clinical/human studies. Considering the fact that clove is a GRAS food, it is important that human studies be performed and documented. More studies on bioavailability, accumulation, toxicity, dosage and efficacy of clove as a spice drug or functional foods in biological systems especially humans are required. Further, many applications of clove in the food, health, cosmetics, pharmaceutics, nanoparticles, and agricultural industries are still open for investigations.
